# Spectrum-efficient user grouping and resource allocation based on deep reinforcement learning for mmWave massive MIMO-NOMA systems

**DOI:** 10.1038/s41598-024-59241-x

**Published:** 2024-04-17

**Authors:** Minghao Wang, Xin Liu, Fang Wang, Yang Liu, Tianshuang Qiu, Minglu Jin

**Affiliations:** 1 College of Electronic Information Engineering, Inner Mongolia University, Hohhot, 010021 China; 2 Faculty of Electronic Information and Electrical Engineering, Dalian University of Technology, Dalian, 116024 China

**Keywords:** Engineering, Electrical and electronic engineering

## Abstract

Millimeter-wave (mmWave) massive multiple-input multiple-output non-orthogonal multiple access (MIMO-NOMA) is proven to be a primary technique for sixth-generation (6G) wireless communication networks. However, the great increase in users and antennas brings challenges for interference suppression and resource allocation for mmWave massive MIMO-NOMA systems. This study proposes a spectrum-efficient and fast convergence deep reinforcement learning (DRL)-based resource allocation framework to optimize user grouping and allocation of subchannel and power. First, an enhanced K-means grouping algorithm is proposed to reduce the multi-user interference and accelerate the convergence. Then, a dueling deep Q-network (DQN) structure is proposed to perform subchannel allocation, which further improves the convergence speed. Moreover, a deep deterministic policy gradient (DDPG)-based power resource allocation algorithm is designed to avoid the performance loss caused by power quantization and improve the system’s achievable sum-rate. The simulation results demonstrate that our proposed scheme outperforms other neural network-based algorithms in terms of convergence performance, and can achieve higher system capacity compared with the greedy algorithm, the random algorithm, the RNN algorithm, and the DoubleDQN algorithm.

## Introduction

There is a dramatic increase in the users and requirements for the corresponding wireless data traffic in the fifth-generation (5G) and sixth-generation (6G) mobile communication networks^1^. The millimeter-wave (mmWave) massive multiple-input multiple-output (MIMO), which provides greater bandwidth and higher multiplexing gain to enhance the capacity and effectiveness of the wireless communication systems, has been considered to be effective for resolving the above issue^2,3^. To further improve the capacity and utilization efficiency of time-frequency resource of the network^4,5^, non-orthogonal multiple access (NOMA) is introduced into mmWave massive MIMO system^6–8^. However, with the explosive deployment of base stations (BSs) and ultra interconnections of user terminals, the number of links and data transmission rates between the BS and users increase significantly, then the wireless resources of the mmWave massive MIMO-NOMA network become more relatively limited. Moreover, due to the dynamic changes of the system, the existing traditional resource allocation schemes without self-adaptability and autonomous decision-making will limit the performance improvement. Therefore, it is crucial to optimize the resource allocation for maximizing the data rate in mmWave massive MIMO-NOMA systems.

The resource allocation is typically formulated and solved using optimization methods^9^. The traditional exhaustive search method, greedy algorithm^10^, fractional programming algorithm^11^, quality-of-service (QoS)-oriented algorithm^12^, branch-and-bound method^13^, and successive convex approximation based suboptimal scheme^14^ are exploited to obtain optimal or sub-optimal solutions when the optimization problem is non-convex, resulting in high computational complexity. Compared with these existing traditional technologies, machine learning (ML) can be exploited online for optimizing resource allocation with reduced complexity^15^. Many researchers have conducted various studies on data-driven deep learning (DL) based and reinforcement learning (RL) based resource allocation techniques. Sher et al.^16^ proposed a method for joint resource allocation and RRH association in multi-tier 5G networks, which leverages DL to develop resource allocation techniques, optimize RA decisions, and alleviate excessive computational burden. In addition, Luo et al.^17^ explored DL applications in user association, subchannel allocation, and power optimization for NOMA networks, which integrates supervised and semi-supervised learning to enhance energy efficiency in complex wireless communication scenarios. However, one drawback of these methods is the need for abundant labeled data for efficient and computationally expensive model training, which will incur significant computational costs in large-scale cellular networks. Moreover, dealing with hidden variables can introduce inaccuracies, further diminishing the effectiveness of these techniques^18^.

By using agent to learn the optimal allocation policy, RL has great potential for wireless resource allocation. Luo et al.^19^ adopted a mutual Q-network to solve the power allocation for improving the spectrum efficiency of the wireless network. In the previous study^20^, a state-action-reward-state-action (SARSA) power allocation algorithm was proposed based on traditional RL to increase the average throughput. However, the value of the Q-function estimated by traditional value-based algorithms, including SARSA and Q-learning based algorithms, must be determined to obtain the optimal strategy^21^. Specifically, traditional Q-learning requires traversing and evaluating each state-action pair, resulting in low convergence speed and high computational complexity^22^. Therefore, the traditional Q-learning method is not feasible in large-scale cellular networks that exhibit high-dimensional state and action spaces. Mezzavilla et al.^23^ used a neural network to deal with this problem and approximate the Q-function value for a mmWave cellular network. RL is unstable and even diverges when estimating Q-function value by neural network due to the existing correlation between training data. In addition, deep reinforcement learning (DRL) has been proven to be a new technique for intelligent resource allocation in communication cellular networks by using the perceptual capabilities of DL and the decision-making capabilities of RL^24^. A previous study^25^ developed a DRL method to obtain the optimal value of the Q-function by integrating the classical Q-learning with a deep neural network (DNN), which aims to address the above problems of traditional RL. The new proposed deep Q-network (DQN) structure makes the training model more efficient and stable by introducing an experience replay mechanism that can disrupt the correlation between data.

Recently, data-driven model-free DRL methods have attracted tremendous attention for solving resource management in cellular networks^26–33^. Naparstek et al.^26^ allocated the dynamic spectrum and maximized network utility of the wireless network by DRL. Xu et al.^27^ effectively minimized the total power consumption and ensured the users demand by a proposed deep Q-learning (DQL)-based power resources allocation algorithm. Zhao et al.^28^ solved the joint user association and resource allocation based on the DRL for the downlink of a heterogeneous network (HetNets). This scheme aims to optimize the long-term utility of the network and simultaneously guarantee the QoS requirements. Nasir et al.^29^ utilized the multi-agent DQL to allocate power and improve spectral efficiency of the wireless network. Compared with single resource allocation, the joint resource allocation can improve resource utilization and throughput. Liang et al.^30^ improved both the bandwidth utilization and energy efficiency for a networking graph-based Internet-of-Things (IoT) system with DQN. Huang et al.^31^ proposed a cooperative multi-agent RL framework with Double DQN for allocating the power resources and discrete spectrum. However, traditional DQN and Double DQN have the problem with slow convergence speed in high-dimensional action space due to the discrete power actions. Thus, Cao et al.^32^ combined DQN with back propagation neural network (BPNN) to allocate resources of a MIMO-NOMA system, which substantially improves bandwidth utilization efficiency. Unfortunately, searching results in the quantized power set during the training of BPNN causes an increase in computational complexity. Wang et al.^33^ attempted to jointly allocate the subchannel and power resources with DRL for a NOMA cellular network. Guo et al.^34^ advanced this field by developing the multi-agent proximal policy optimization (PPO) algorithm, a multi-agent DRL approach designed to refine handover control and power allocation in HetNets. This method features centralized training for decentralized user equipment policies, enhanced by a counterfactual baseline to address credit assignment challenges. Despite PPO’s benefits, its limitations prompted the development of the A3C algorithm. Complementing these efforts, Sun et al.^35^ introduced a dual-layer Asynchronous Advantage Actor-Critic (A3C)-based optimization algorithm, focusing on residual resources like offloading ratio and transmission power, and innovated with a swap-matching algorithm for superior subchannel allocation, thereby providing new insights into the field of resource allocation. Although these schemes improve the energy efficiency of the network, it does not consider multi-user and co-channel interference in NOMA. The existing resource allocation optimizations by applying DRL technology are not appropriate to be applied to mmWave massive MIMO-NOMA systems. It is extremely worthy of further exploration of optimizations for resource allocation.

In this study, we investigate the user grouping, subchannel, and power allocation optimization problem in a downlink multi-user mmWave massive MIMO-NOMA system. First, a new initial cluster center selection method is designed for user grouping, which reduces the interference of the system and accelerates the convergence speed. Then, for quickly determining the optimal resource allocation and improving the system spectral efficiency, a joint framework based on dueling DQN and deep deterministic policy gradient (DDPG) is designed. Specifically, we use dueling DQN for subchannel allocation network to further improve the convergence speed and employ DDPG to perform continuous power allocation and avoid the performance degradation caused by power quantization errors in DQN. The experimental results demonstrate that the proposed scheme exhibits fast can accelerate the convergence speed of resource allocation and effectively improves the spectral efficiency of the system. The contributions of this study are summarized as follows:

1. An optimization model for user grouping, subchannel, and power allocation under reasonable constraints for a spectral-efficient mmWave massive MIMO-NOMA system is formulated.
2. We propose an enhanced K-means user grouping scheme for the system, in which the initial cluster center is selected based on channel gain and channel correlation rather than randomly selection to accelerate the convergence speed of user grouping. Subsequently, users with weak correlation are assigned to different groups to alleviate multi-user interference.
3. In addition, a subchannel allocation algorithm based on dueling DQN is proposed, which uses an improved Q-network to efficiently estimate the state values and the state-dependent actions. Because the Q-value of each action is obtained under the advantage state, the optimal subchannel allocation action can be achieved with high convergence speed.
4. Moreover, a DDPG-based continuous power allocation scheme on the corresponding subchannel is proposed to obtain the optimal power allocation action, which utilizes random weights and the current state to generate a deterministic power allocation action, then adjusts the weights by a soft update method to obtain a target power allocation action and a target Q-network. The proposed power allocation scheme efficiently handles a continuous power action space and avoids power quantization errors caused by discrete actions, thus further improving the spectral efficiency of the system.

The remainder of this paper is organized as follows: Section “System model and problem formulation” introduces the mmWave massive MIMO-NOMA system model and problem formulation. Section “User grouping and dueling DQN-DDPG based resource allocation scheme” presents the proposed joint resource allocation scheme for the mmWave massive MIMO-NOMA system. The simulation results are presented and discussed in Section “Simulation results”. Finally, this study is concluded in Section “Conclusion”.

*Notation*: The identity matrix of size $N \times N$ is denoted as ${{\textbf{I}}_N}$. ${\left( \cdot \right) ^H}$ denotes the conjugate transpose operation. $[ {\textbf{A}} ]_{:,i}$ and $[ {\textbf{A}} ]_{i,j}$ denote the *i*th column and $\left( {i,j} \right)$th element of ${\textbf{A}}$, respectively. $[ {\textbf{a}} ]_i$ and $\left| a \right|$ denote the *i*th element of vector ${\textbf{a}}$ and the absolute value of *a*, respectively. $\left| {{{\mathscr {A}}}} \right|$ denotes the number of elements in set ${{\mathscr {A}}}$. Function ${{\mathbb {E}}}\left( \cdot \right)$ provides the expectation operation.

## System model and problem formulation

### System model

**Figure 1:**
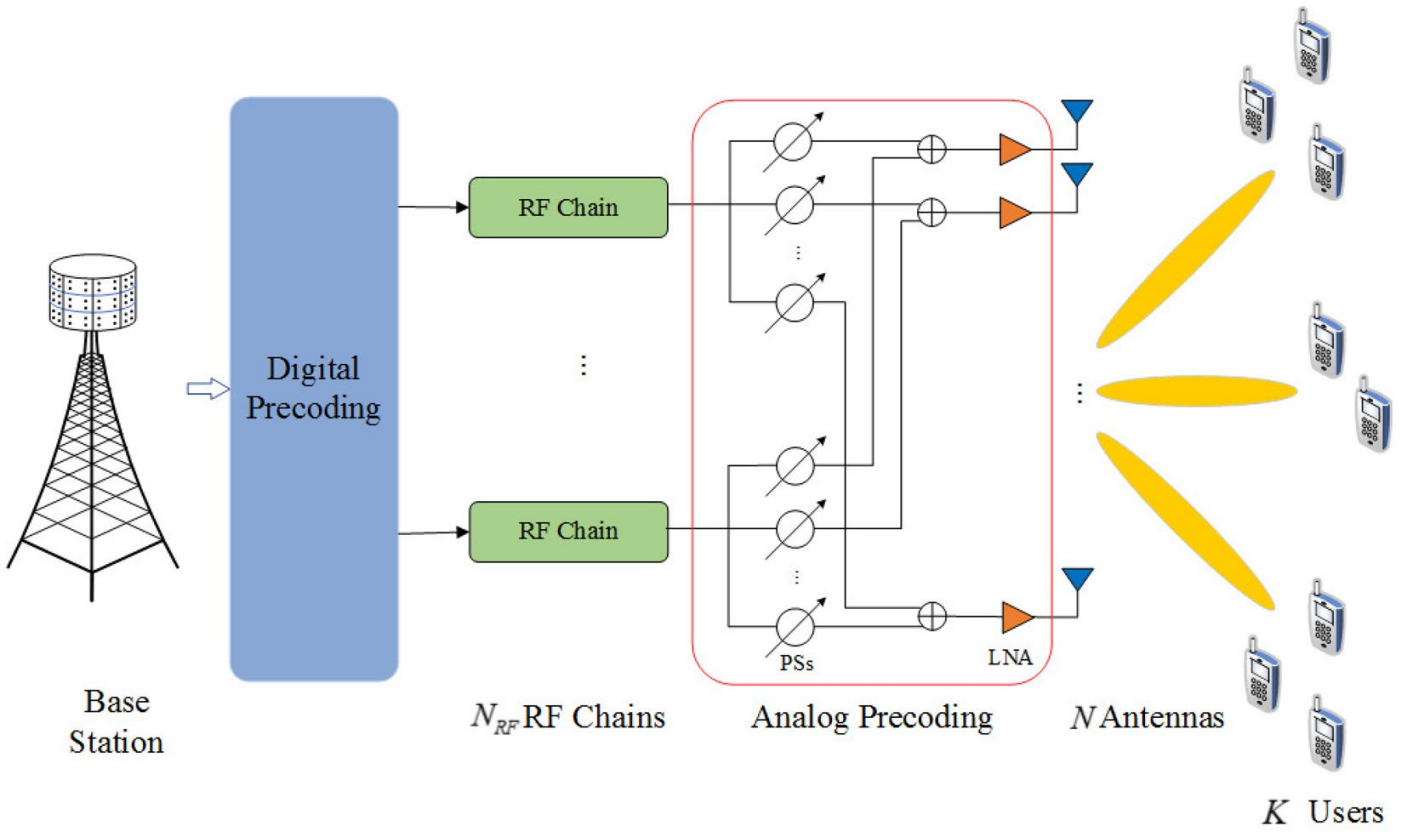
Diagram of a downlink mmWave massive MIMO-NOMA system.

A single-cell multi-user mmWave massive MIMO-NOMA system for downlink transmission that equips a BS with $N_a$ antennas and ${N_{RF}}$ radio frequency (RF) chains that serves *K* single-antenna users is investigated. The system is depicted in Fig. 1. Due to the hardware limitations, only a small number of RF chains is deployed at the BS (i.e., ${N_{RF}} < N_a$). To achieve a high multiplexing gain, the number of data streams is set as the number of RF chains (i.e., ${N_s} = {N_{RF}}$). The *K* users are then divided into ${N_{RF}}$ groups by the user grouping method, and a separate data stream is corresponded to one group. ${\Omega _m}$ is denoted as the set of users in the *m*th group, and $\left| {{\Omega _m}} \right|$ denotes the number of users which is guaranteed to be at least 1 (i.e., $\left| {{\Omega _m}} \right| \ge 1$) to ensure that the RF resources are not left idle. In addition, there will be no overlap between different user groups, i.e., ${{\Omega }_{i}}\bigcap {{\Omega }_{j}}= \varnothing$ and $\sum \limits _{m = 1}^{{N_{RF}}} {\left| {{\Omega _m}} \right| } = K$, where $\varnothing$ denotes the empty set. This means that each user belongs to only one group and not multiple groups at the same time, which can avoid multi-user interference among users. The set of users is expressed as $\Omega = \left\{ {{\Omega _1},{\Omega _2}, \ldots ,{\Omega _{{N_{RF}}}}} \right\}$ after user grouping. To avoid the interference between subchannels, the system bandwidth is divided into *L* orthogonal subchannels, and the subchannels set is represented as ${{{\mathscr {L}}}}= \left\{ {1,2, \ldots ,L} \right\}$. The received signal for the *k*th user in the *m*th group on the *l*th subchannel is expressed as

1 $$\begin{aligned} {y_{l,m,k}} = {\textbf{h}}_{l,m,k}^H{\textbf{WPs}} + {n_{l,m,k}}, \end{aligned}$$

where the rapidly-varying channel state information (CSI) ${{\textbf{h}}_{l,m,k}}$ is assumed to be instantaneously known to both the BS and UEs and satisfies ${{\textbf{h}}_{l,m,k}} \in {{{\mathbb {C}}}^{N_a \times 1}}$^36^. In addition, ${\textbf{s}}$ is the $K \times 1$ original transmit signal vector with normalized power ${{\mathbb {E}}}\left( {{\textbf{s}}{{\textbf{s}}^H}} \right) = {{\textbf{I}}_K}$. The ${N_{RF}} \times K$ power allocation matrix is ${\textbf{P}}=dia g\left( {{{\textbf{p}}_1},{{\textbf{p}}_2}, \ldots ,{{\textbf{p}}_{{N_{RF}}}}} \right)$, in which ${{\textbf{p}}_m}=\left[ {\sqrt{{P_{l,m,1}}} ,\sqrt{{P_{l,m,2}}} , \ldots ,\sqrt{{P_{l,m,k}}} ,\ldots ,\sqrt{{P_{l,m,\left| {{\Omega _m}} \right| }}} } \right] ^T$, where ${P_{l,m,k}}$ denotes the allocated transmit power of the *k*th user in the *m*th group of the *l*th subchannel. Hybrid full-connected precoding is used on the BS, and the precoding matrix is denoted as ${\textbf{W}} = {\textbf{AD}} = [{{\textbf{w}}_1},{{\textbf{w}}_2}, \ldots ,{{\textbf{w}}_{{N_{RF}}}}]$, where ${\textbf{A}}$ and ${\textbf{D}}$ are the analog and digital precoding matrices, respectively. In general, each column element of ${\textbf{W}}$ is assumed to satisfy $\left\| {{{\textbf{w}}_m}} \right\| = 1$, $1 \le m \le {N_{RF}}$. Moreover, ${n_{l,m,k}}$ is the additive Gaussian white noise with variance ${ \sigma _{n}^{\textrm{2}}}$.

A subchannel allocation factor ${x_{l,m,k}}\left( t \right)$ is used to indicate the allocation of the subchannel at the *t*th time slot ($l \in {{{\mathscr {L}}}}$). When the *l*th subchannel is allocated to the *k*th user in the *m*th group at the *t*th time slot, ${x_{l,m,k}}\left( t \right) = 1$. Otherwise, ${x_{l,m,k}}\left( t \right) {\mathrm{= 0}}$. At the beginning of a time slot, the instantaneous channel gain is available to the BS and it does not change during the time slot^37,38^. Then, the received signal of the *k*th user in the *m*th group of the *l*th subchannel at the *t*th time slot is given by

2 $$\begin{aligned} \begin{aligned} {y_{l,m,k}}\left( t \right)&={x_{l,m,k}}\left( t \right) {\textbf{h}}_{l,m,k}^H\left( t \right) {{\textbf{W}}_m}\left( t \right) \sqrt{{P_{l,m,k}}\left( t \right) } {s_{l,m,k}}\left( t \right) \\&\quad +\sum \limits _{i = 1,i \ne k}^{\left| {{\Omega _m}} \right| } {x_{l,m,i}\left( t \right) } {\textbf{h}}_{l,m,i}^H\left( t \right) {{\textbf{W}}_m}\left( t \right) \sqrt{{P_{l,m,i}}\left( t \right) } {s_{l,m,i}}\left( t \right) + {n_{l,m,k}}\left( t \right) \\&= {x_{l,m,k}}\left( t \right) {g_{l,m,k}}\left( t \right) \sqrt{{P_{l,m,k}}\left( t \right) } {s_{l,m,k}}\left( t \right) \\&\quad +\sum \limits _{i = 1,i \ne k}^{\left| {{\Omega _m}} \right| } {x_{l,m,i}\left( t \right) } {g_{l,m,i}}\left( t \right) \sqrt{{P_{l,m,i}}\left( t \right) } {s_{l,m,i}}\left( t \right) + {n_{l,m,k}}\left( t \right) , \end{aligned} \end{aligned}$$

where ${\textbf{h}}_{l,m,k}^H\left( t \right) {{\textbf{W}}_m}\left( t \right) = {g_{l,m,k}}\left( t \right)$ represents the effective channel gain. The original transmit signal of the *i*th user in the *m*th group on the *l*th subchannel ${s_{l,m,i}}$ ($k + 1 \le i \le \left| {{\Omega _m}} \right|$) can be decoded by the *k*th user in the *m*th group on the *l*th subchannel. And then it is removed from the received signal in a successive manner. The optimal decoding order is the increasing order of user’s effective channel gain according to the NOMA rules^5^; thus, it is assumed that the order of the effective channel gains of the users in the *m*th group of the *l*th subchannel is ${\left| {{\textbf{h}}_{l,m,1}^H{{\textbf{W}}_m}} \right| ^2} \ge {\left| {{\textbf{h}}_{l,m,2}^H{{\textbf{W}}_m}} \right| ^2} \ge \ldots \ge {\left| {{\textbf{h}}_{l,m,\left| {{\Omega _m}} \right| }^H{{\textbf{W}}_m}} \right| ^2}$^6^. The signal-to-interference-plus-noise ratio (SINR) of the *k*th user in the *m*th group on the *l*th subchannel at the *t*th time slot is defined as

3 $$\begin{aligned} \begin{aligned} {\textrm{SIN}}{{\textrm{R}}_{l,m,k}}\left( t \right) =\frac{{{x_{l,m,k}}\left( t \right) {{\left| {{\textbf{h}}_{l,m,k}^H\left( t \right) {{\textbf{W}}_m}\left( t \right) } \right| }^2}{P_{l,m,k}}\left( t \right) }}{{{x_{l,m,k}}\left( t \right) {{\left| {{\textbf{h}}_{l,m,k}^H\left( t \right) {{\textbf{W}}_m}\left( t \right) } \right| }^2}\sum \limits _{j = 1}^{k - 1} {{P_{l,m,j}}} \left( t \right) +\sum \limits _{i \ne m} {\sum \limits _{k = 1}^{\left| {{\Omega _i}} \right| } {{x_{l,m,i}}\left( t \right) {{\left| {{\textbf{h}}_{l,m,k}^H\left( t \right) {{\textbf{W}}_i}\left( t \right) } \right| }^2}{P_{l,i,k}}} } \left( t \right) + {\sigma _{n}^{\textrm{2}}}}}, \end{aligned} \end{aligned}$$

In this way, the data rate of the *k*th user is expressed as

4 $$\begin{aligned} {R_{l,m,k}}\left( t \right) =B_l{\log _2}\left( {1 + {{{\textrm{SINR}} }_{l,m,k}}\left( t \right) } \right) , \end{aligned}$$

where $B_l$ is the subchannel bandwidth and $B_l= B/L$ with *B* being the available system bandwidth. The achievable sum-rate is given by

5 $$\begin{aligned} {R_{sum}}\left( t \right) = \sum \limits _{l = 1}^L {\sum \limits _{m = 1}^{{N_{RF}}} {\sum \limits _{k = 1}^{\left| {{\Omega _m}} \right| } {{R_{l,m,k}}} } } \left( t \right) . \end{aligned}$$

### Channel model

The widely used geometric channel model is used and the uniform linear array (ULA) with a half-wavelength antenna space is employed in BS^39^. The multipath channel between the BS and the *k*th user in the *m*th group on the *l*th subchannel is given by

6 $$\begin{aligned} {{\textbf{h}}_{l,m,k}} = \sqrt{\frac{N_a}{{{F_{l,m,k}}}}} \sum \limits _{f = 1}^{{F_{l,m,k}}} {\alpha _{_{l,m,k}}^f} {{\textbf{a}}^H}\left( {\theta _{_{l,m,k}}^f} \right) , \end{aligned}$$

where $\alpha _{l,m,k}^f$ represents the complex path gain of the *f*th path, which follows the Rayleigh distribution with zero mean and variance $\sigma ^{2}$. $\theta _{l,m,k}^f \in \left[ {0,2\pi } \right]$ is the azimuth angle of departure of the *f*th path, and ${F_{l,m,k}}$ is the total number of multipath components. ${\textbf{a}}\left( {\theta _{l,m,k}^f} \right)$ is the corresponding antenna array steering vector in the direction of ${\theta _{l,m,k}}$, which is expressed as

7 $$\begin{aligned} {\textbf{a}}\left( {\theta _{_{l,m,k}}^f} \right) = \sqrt{\frac{1}{N_a}} {\left[ {1,{e^{j\frac{{2\pi }}{\lambda }d\sin \left( {\theta _{l,m,k}^f} \right) }},\ldots , {e^{j\frac{{2\pi }}{\lambda }\left( {N - 1} \right) d\sin \left( {\theta _{l,m,k}^f} \right) }}}\right] ^{T}}, \end{aligned}$$

where $\lambda$ and *d* are the wavelength and antenna space, respectively.

### Problem formulation

There are some kinds of resources that need to be utilized effectively in mmWave massive MIMO-NOMA systems, such as channel, time slot, bandwidth, beam, power and so on. Because the demand for mobile data rate increases explosively, channel allocation and power allocation are extremely significant in radio resource management^40,41^. The maximization of achievable sum-rate under constraints of user minimum data rate and the BS maximum transmit power through choosing the subchannel allocation factor ${x_{l,m,k}}\left( t \right)$ and the allocated power ${P_{l,m,k}}\left( t \right)$ for all users is the optimization objective, which is expressed to

8 $$\begin{aligned} \begin{aligned} \mathop {\max }\limits _{{x_{l,m,k}}(t),{P_{l,m,k}}(t)}&\quad \sum \limits _{l = 1}^L {\sum \limits _{m = 1}^{{N_{RF}}} {\sum \limits _{k = 1}^{\left| {{\Omega _m}} \right| } {{R_{l,m,k}}\left( t \right) } } } \\&\quad = \sum \limits _{l = 1}^L {\sum \limits _{m = 1}^{{N_{RF}}} {\sum \limits _{k = 1}^{\left| {{\Omega _m}} \right| } B{\log _2 \left( {1 + {{{\textrm{SINR}} }_{l,m,k}}\left( t \right) } \right) } } },\\ s.t.&\quad C1: \sum \limits _{l = 1}^L {\sum \limits _{m = 1}^{{N_{RF}}} {\sum \limits _{k = 1}^{\left| {{\Omega _m}} \right| } {{P_{l,m,k}}} \left( t \right) \le {P_{\max }}} };\\&\quad C2:{P_{l,m,k}}\left( t \right) \ge 0, \forall l,m,k;\\&\quad C3:{R_{l,m,k}}\left( t \right) \ge {R_{\min }},\forall l,m,k;\\&\quad C4:{x_{l,m,k}}\left( t \right) \in \left\{ {0,1} \right\} ,\forall l,m,k;\\&\quad C5:1 \le \sum \limits _{k = 1}^{\left| {{\Omega _m}} \right| } {{x_{l,m,k}}\left( t \right) \le C}, \forall l,m;\\&\quad C6:\sum \limits _{l = 1}^L {{x_{l,m,k}}\left( t \right) \le 1}, \forall m,k. \end{aligned} \end{aligned}$$

C1 limits the total transmit power ${P_{\max }}$ cannot be exceeded. Constraint C2 guarantees non-negative of the allocated power for each user. Constraint C3 demonstrates that the data rate of any user is not less than the minimum threshold ${R_{\min }}$ to ensure the QoS requirements for users^6^. Constraint C4 is the current subchannel allocation, where ${x_{l,m,k}}\left( t \right) = 1$ and ${x_{l,m,k}}\left( t \right) {\mathrm{= 0}}$ indicate that the current subchannel resource is occupied and idle, respectively. Constraint C5 indicates that each subchannel can support at least one user and no more than *C* users at a time, where *C* is set to 4 following^42^. Constraint C6 restricts that each user can only occupy at most one subchannel, which can avoid power allocation over subchannels in the situation of each user occupying multiple subchannels simultaneously^8^.

Traditional heuristic or alternating iterative methods solve the non-convex and NP-hard joint optimization problem (Eq. 8) with unaffordable complexity and limited performance. Contrary, DRL can reduce computational complexity and improve system performance by fully exploring the hidden information of big data and dynamically real-time interacting with wireless communications networks^6^. Therefore, we make better use of DRL to solve the non-convex and NP-hard problem of Eq. (8).

## User grouping and dueling DQN-DDPG based resource allocation scheme

The non-convex and NP-hard optimization problem (Eq. 8) is difficult to be solved by traditional optimization methods, which results in slow convergence and low system spectral efficiency. Therefore, we propose to use dueling DQN-DDPG to address this problem for mmWave massive MIMO-NOMA system.

### User grouping

Owing to the high directionality and the large number of users of the mmWave massive MIMO-NOMA system (i.e., $K > {N_{RF}}$), user grouping is vital to increase the multiplexing gain and reduce the interference. Since the beam pattern of users in a group is the same and the distinguished beams of different groups are different, an enhanced K-means user grouping algorithm is proposed. Specifically, users with strong channel correlation are assigned to the same group, which increases multiplexing gain. Accordingly, to reduce interference, users with weak correlation are assigned to different groups. The normalized channel correlation between two users can be calculated by

9 $$\begin{aligned} {C_{i,j}} = \frac{{\left| {{\textbf{h}}_i^H{{\textbf{h}}_j}} \right| }}{{{{\left\| {{{\textbf{h}}_i}} \right\| }_2}{{\left\| {{{\textbf{h}}_j}} \right\| }_2}}}. \end{aligned}$$

Although K-means clustering algorithm is widely used for performing user grouping, the initial cluster center is selected randomly by traditional K-means clustering algorithm, resulting in high computation and slow convergence rate^6^. To solve the poor quality of clusters, we design a new initial cluster center selection method as follows:

1. Select the user with the highest channel gain as the first cluster center $\Gamma _1$.
2. Select the user with the lowest channel correlation with the first cluster center as the center of the second cluster $\Gamma _2$. The channel correlation between the users and the first cluster center is calculated according to Eq. (9).
3. Calculate the channel correlation of all users with the nearest cluster center and select the user with the largest correlation as the new cluster center $\Gamma _m$.
4. If $m=N_{RF}$, $N_{RF}$ cluster centers have been selected, stop the iteration. Otherwise, repeat step 3 until $N_{RF}$ cluster centers are selected.
5. Return the set of selected $N_{RF}$ cluster centers $\left\{ {{\Gamma _1},{\Gamma _2}, \ldots ,{\Gamma _{{N_{RF}}}}} \right\}$.

The new initial cluster center selection method is shown in Algorithm 1.

**Algorithm 1:**
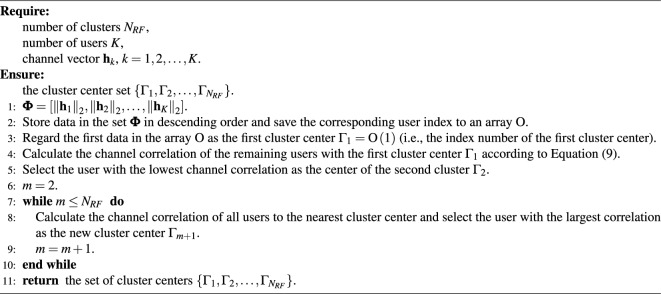
The initial cluster center selection algorithm

Subsequently, the other users with the strongest channel correlation to the cluster centers are assigned to the corresponding cluster. The assignment of the *k*th user to the *M*th cluster is indicated as

10 $$\begin{aligned} M = \mathop {\arg \max }\limits _{1 \le m \le {N_{RF}}} {C_{k,{\Gamma _m}}}. \end{aligned}$$

The correlation between the user and users in other clusters is defined as

11 $$\begin{aligned} {C_k} = \sum \limits _{1 \le j \le K}^{j \notin {\Omega ^{\left( k \right) }}} {{C_{k,j}}}, \end{aligned}$$

where ${\Omega ^{\left( k \right) }}$ is the cluster with user *k*. The user with the lowest correlation to users in other clusters is updated to be the new center of each cluster, which can further reduce the channel correlation between different clusters. The center of the *m*-cluster is updated as

12 $$\begin{aligned} {\Gamma _m}: = \mathop {\arg \min }\limits _{1 \le k \le \left| {{\Omega _m}} \right| } {C_k}, \end{aligned}$$

where ${\Omega _m}$ is the *m*th cluster, $m= 1,2, \ldots ,{N_{RF}}$. The iteration stops when the center of the cluster does not change. The proposed user-grouping algorithm is presented in Algorithm 2.

**Algorithm 2:**
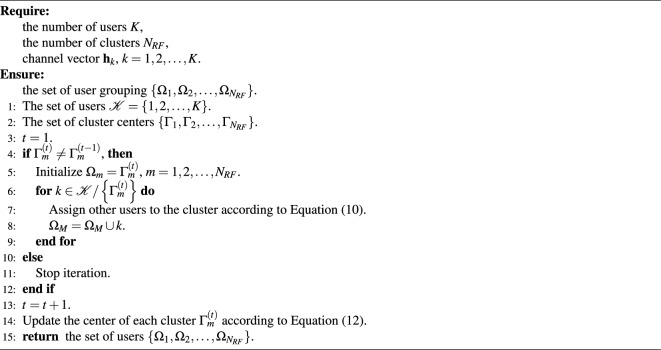
Proposed user grouping algorithm

### Proposed dueling DQN-DDPG based resource allocation scheme

To reduce the impact of multipath fading on the transmission quality of subchannels and the achievable sum-rate of system, we have proposed to jointly assign subchannels and allocate power to receivers in each user group based on traditional DQN network, as shown in Appendix A. However, traditional DQN does not fully represent the Q value in practice because of its discreteness, resulting in slow convergence. Moreover, due to the missing information of quantization and high computational complexity caused by high quantization level, power quantization can cause performance loss. Thus, we propose an enhanced resource allocation scheme to address these problems by exploiting the dueling DQN and DDPG.

**Figure 2:**
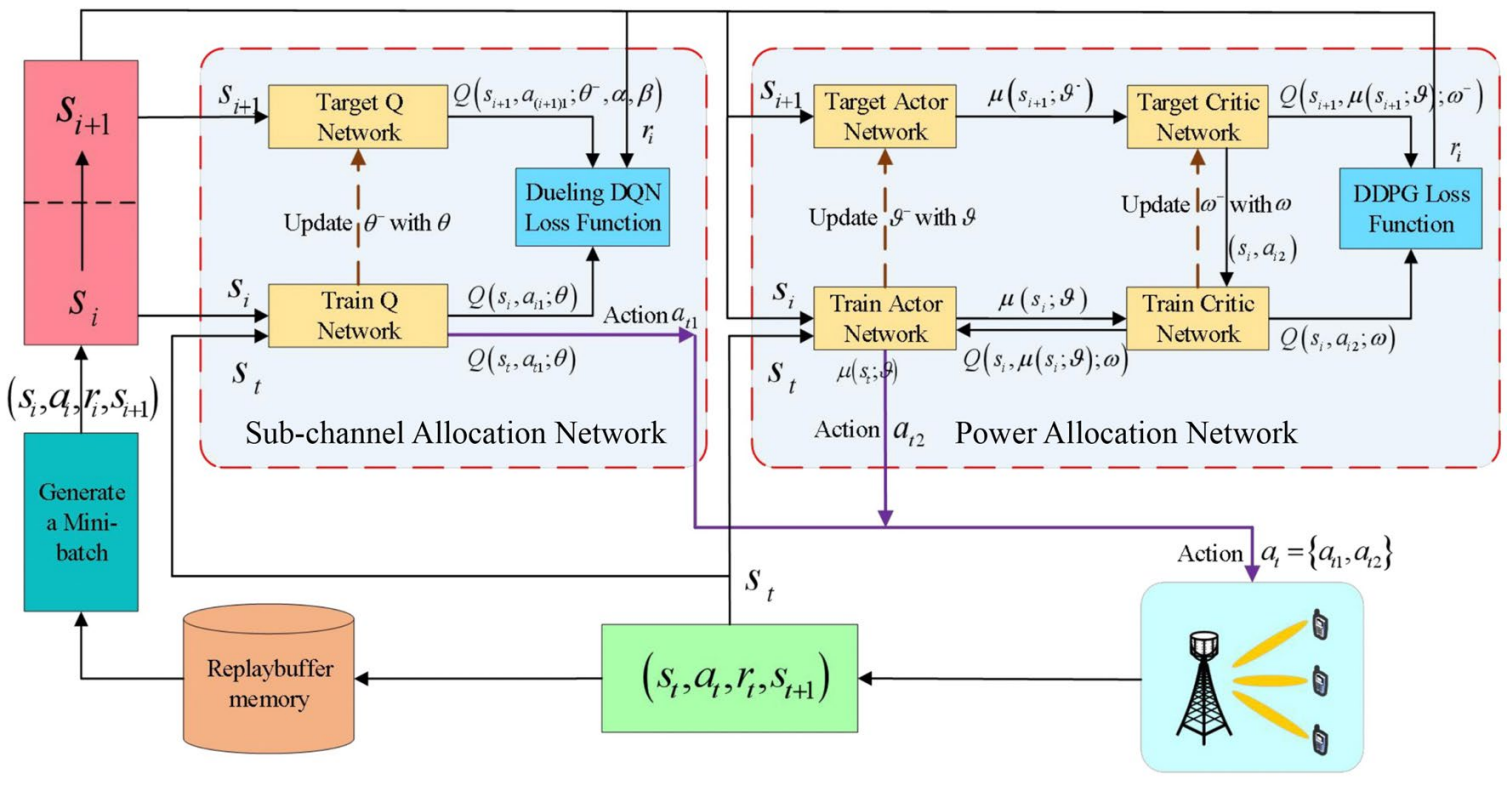
Dueling DQN-DDPG-based subchannel and power resource allocation network.

The proposed dueling DQN-DDPG-based algorithm is shown in Fig. 2, in which state space ${{\mathscr {S}}}$, action space ${{\mathscr {A}}}$, and instant reward ${{{\mathscr {R}}}}$ are included. The current state of the network ${s_t}$ is used as the input to the network, which is obtained by agent through interacting with the network. The network outputs the Q value after the action ${a_t}$ is taken from action space ${{{\mathscr {A}}}}$ by the agent. Then, the agent can receive an instant reward ${r_t}$ from the network and the next state ${s_{t + 1}}$. These components are defined as follows:

1. *States of dueling DQN-DDPG*: State ${s_t} \in {{{\mathscr {S}}}}$ needs to reflect the current SINR of all users. The proposed method can learn the performance of all possible configurations, and once learned, it can provide the best configuration for every given CSI. Therefore, to get the SINR of all users and make allocation decisions, each user has to report some effective information to BS through the feedback link, including channel information ${{\textbf{h}}_{l,m,k}}$ of *k*th user in the *m*th group of *l*th subchannel, hybrid precoding matrix ${\textbf{W}}_m$ in the *m*th group and the interference information. Then, the system can obtain SINR from the feedback information according to Eq. (3). Therefore, the states at the *t*th time slot are defined as
  13 $$\begin{aligned} {s_t} = \left\{ {{\textrm{SIN}}{{\textrm{R}}_{l,m,k}}(t)} \right\} . \end{aligned}$$
2. *Actions of dueling DQN-DDPG*: The action space ${{{\mathscr {A}}}}$ contains all the available subchannel allocation factors ${x_{l.m.k}}\left( t \right)$ and the allocated power ${P_{l,m,k}}\left( t \right)$. Thus, the actions at the *t*th time slot are defined as
  14 $$\begin{aligned} {a_t} = \left\{ {{a_{t1}},{a_{t2}}} \right\} , \end{aligned}$$
  where $a_{t1}=\{{x_{1,1,1}}(t),\ldots ,x_{l,m,|{\Omega _m}|}(t),\ldots ,x_{L,{N_{RF},|{\Omega _{{N_{RF}}}}|}}(t)\}$, $a_{t1}\in {{{{\mathscr {A}}}}_1}$. The DDPG network generates a deterministic power allocation action ${a_{t2}} = \mu \left( {{s_t};\vartheta } \right)$ according to the random weights $\vartheta$ and the current state ${s_t}$ for the power allocation network. A random exploration noise is introduced to balance the exploration and exploitation of ${a_{t2}}$, which is defined as
  15 $$\begin{aligned} {a_{t2}} = \mu \left( {{s_t};\vartheta } \right) + {N_t}, \end{aligned}$$
  where ${N_t}$ is the random exploration noise and ${a_{t2}}$ is restricted to the range of $\left[ {0,{P_{\max }}} \right]$. To complete the power allocation, the system needs to get channel information and decide the SINR value in each user group which can be obtained by the result of subchannel allocation. Therefore, it is necessary to allocate the subchannel according to the initial values of the random power allocation factors, and allocate power to receivers in each user group until the subchannel allocation scheme converges.
3. *Reward function of dueling DQN-DDPG*: After selecting action ${a_t}$ in the current state ${s_t}$, we regard the achievable sum-rate as the instant reward ${r_t} \in {{{\mathscr {R}}}}$. By using the constraint of C3, the instant reward ${r_t}$ at the *t*th time slot is defined as
  16 $$\begin{aligned} {r_t} = \left\{ {\begin{array}{ll} {\sum \limits _{l = 1}^L {\sum \limits _{m = 1}^{{N_{RF}}} {\sum \limits _{k = 1}^{\left| {{\Omega _m}} \right| } {{R_{l,m,k}}\left( t \right) ,} } } } &{}\quad {{R_{l,m,k}}\left( t \right) \ge {R_{\min }},\forall l,m,k} \\ {0,} &{}\quad {{\textrm{otherwise}}} \\ \end{array}} \right. . \end{aligned}$$
  Then, the long-term cumulative discounted reward function ${{{{\mathscr {W}}}}_t}$ at the *t*th time slot can be expressed as
  17 $$\begin{aligned} \begin{aligned} {{{{\mathscr {W}}}}_t}&= \sum \limits _{i = 0}^\infty {{\gamma ^i}} {r_{t + i}} \\&= \sum \limits _{i = 0}^\infty {{\gamma ^i}} \sum \limits _{l = 1}^L {\sum \limits _{m = 1}^{{N_{RF}}} {\sum \limits _{k = 1}^{\left| {{\Omega _m}} \right| } {{R_{l,m,k}}\left( {t + i} \right) } } }, \end{aligned} \end{aligned}$$
  where $\gamma \in \left[ {0,1} \right]$ is the discount factor that determines the compromise between the current and predicted future rewards. A larger $\gamma$ indicates that the agent focuses more on the predicted future rewards. From Eq. (16), the agent can obtain more reward when it selects an allocation action that brings a higher achievable sum-rate. Conversely, the agent receives a zero reward if the selected action does not satisfy the constraint of the minimum data rate ${R_{\min }}$.
4. *Training of dueling DQN-DDPG*: The experience replay mechanism is employed to train the proposed dueling DQN-DDPG-based resource allocation scheme. There are two networks in the proposed subchannel allocation scheme, i.e., Q-network $Q\left( {{s_t},{a_t};\theta } \right)$ and target Q-network $Q\left( {{s_{i + 1}},{a_{i + 1}};{\theta ^ - }} \right)$. The Q-network estimates the Q value of the selected action, and the target Q-network generates the target Q value for training. $\theta$ is instantly updated, while ${\theta ^ - }$ is updated with parameter $\theta$ at every *W* time slot. The proposed power allocation network contains four components, including the actor network $\mu \left( {s;\vartheta } \right)$, critic network $Q\left( {s,a;\omega } \right)$, actor target network $\mu \left( {s;{\vartheta ^ - }} \right)$, and the critic target network $Q\left( {s,\mu \left( {s;\vartheta } \right) ;{\omega ^ - }} \right)$. $\vartheta$ and $\omega$ are the weight parameters. $\mu \left( {s;\vartheta } \right)$ and $Q\left( {s,a;\omega } \right)$ are used to select the action and estimate the Q value of the selected action, respectively. And the corresponding target networks $\mu \left( {s;{\vartheta ^ - }} \right)$ and $Q\left( {s,\mu \left( {s;\vartheta } \right) ;{\omega ^ - }} \right)$ are used to generate the training data, where ${\vartheta ^ - }$ and ${\omega ^ - }$ are the weight parameters. BS acts as the agent that uses the SINR as the input of the proposed network based on Eq. (13) and then selects the action ${a_t}$ based on Eqs. (14) and (15) with the current state ${s_t}$. The achievable sum-rate which is defined in Eq. (16) is the returned reward. According to the returned reward, the agent adjusts the action strategy to obtain a better achievable sum-rate. The specific steps are depicted as follows:
  **Step 1:** Initialize the states, actions, weight update interval *W*, and replay memory buffer ${{{\mathscr {D}}}}$ with capacity $\left| {{{\mathscr {D}}}} \right|$. The weights of networks $Q\left( {s,a;\theta } \right)$ and $Q\left( {s,a;{\theta ^ - }} \right)$ are initialized with the random weights $\theta$ and ${\theta ^ - } \leftarrow \theta$, respectively. Initialize the parameters of $\mu \left( {s;\vartheta } \right)$ and $Q\left( {s,a;\omega } \right)$ with random weights $\vartheta$ and $\omega$, respectively. In addition, $\mu \left( {s;{\vartheta ^ - }} \right)$ and $Q\left( {s,a;{\omega ^ - }} \right)$ are initialized with parameters ${\vartheta ^ - } \leftarrow \vartheta$ and ${\omega ^ - } \leftarrow \omega$, respectively.
  **Step 2:** At the beginning of each step (episode), BS first gets the initial state ${s_1}$, which is used as the input of the dueling DQN network for each time slot. Then, the dueling DQN network outputs the estimated values for all subchannel allocation action ${a_{t1}} \in {{{{\mathscr {A}}}}_1}$. The subchannel allocation decision action ${a_{t1}}$ is determined by the estimated Q values and the $\epsilon$-greedy strategy after obtaining all the estimated Q values. Specifically, ${a_{t1}}$ is selected randomly from the action space ${{{{\mathscr {A}}}}_1}$ with probability $\epsilon$. Otherwise, ${a_{t1}} = \arg \mathop {\max }\limits _{{a_{t1}} \in {{{{\mathscr {A}}}}_1}} Q\left( {{s_t},{a_{t1}};\theta } \right)$ is selected with probability $1-\epsilon$, where $0<\epsilon <1$ is a hyperparameter that can make the tradeoff between exploration and exploitation and can be updated by
    18 $$\begin{aligned} \epsilon := max(\epsilon \cdot d,\epsilon _{min}), \end{aligned}$$
    where *d* is the exploration rate decay and $\epsilon _{min}$ is the minimum exploration rate.

**Algorithm 3:**
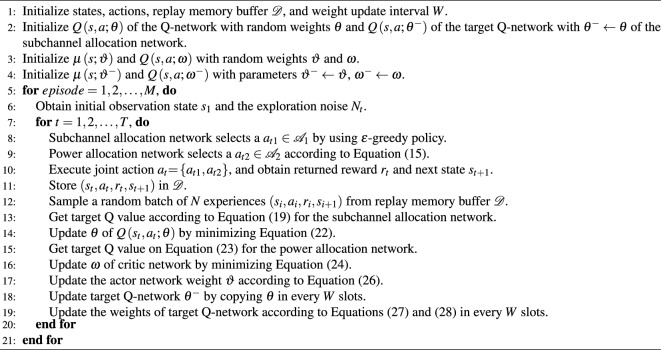
Proposed dueling DQN-DDPG-based scheme for subchannel and power resource allocation
  **Step 3:** In the DDPG network, the deterministic power allocation policy (15) is generated to determine the power allocation decision action ${a_{t2}}$ based on the random weights $\vartheta$, the current state ${s_t}$ and the random exploration noise ${N_t}$.
  **Step 4**: After obtaining the allocation actions ${a_{t1}}$ and ${a_{t2}}$, BS selects the specific subchannel and power for all users according to the joint action ${a_t} = \left\{ {{a_{t1}},{a_{t2}}} \right\}$. Then, BS obtains the returned reward ${r_t}$ by calculating Eq. (16) and receives the next state ${s_{t + 1}}$ from the system.
  **Step 5:** The experience replay mechanism is used to save $\left( {{s_t},{a_t},{r_t},{s_{t + 1}}} \right)$ to ${{{\mathscr {D}}}}$. The data are randomly sampled in the minibatch of batch size *N* from ${{{\mathscr {D}}}}$ to disrupt the correlation between the data and ensure the validity of the training. Then, the target Q-network of the subchannel assignment network generates the target Q value as
    19 $$\begin{aligned} {y_i} = {r_i} + \gamma \mathop {\max }\limits _{{a_{\left( {i + 1} \right) 1}} \in {{{{\mathscr {A}}}}_1}} Q\left( {{s_{i + 1}},{a_{\left( {i + 1} \right) 1}};{\theta ^ - },\alpha ,\beta } \right) . \end{aligned}$$
    The dueling structure splits the Q-function into value function *V* and advantage function *A*, and it has been proven that convergence speed and learning efficiency of the Q value function can be improved by estimating the state values and the state-dependent actions separately^43^. Thus, we define the Q-function of the dueling DQN as
    20 $$\begin{aligned} Q\left( {{s_{i + 1}},{a_{\left( {i + 1} \right) 1}};{\theta ^ - },\alpha ,\beta } \right) =V\left( {{s_{i + 1}};{\theta ^ - },\alpha } \right) +A\left( {{s_{i + 1}},{a_{\left( {i + 1} \right) 1}};{\theta ^ - },\beta } \right) , \end{aligned}$$
    where $\alpha$ and $\beta$ are the parameters of *V* and *A*, respectively. There are infinite possible combinations of functions *V* and *A* for a given Q value if the outputs of the two components are not constrained, however, only a small part of them is reasonable and close to the true values. To solve this problem, the two functions *A* and *V* are restricted as
    21 $$\begin{aligned} Q\left( {{s}_{i+1}},{{a}_{\left( i+1 \right) 1}};{{\theta }^{-}},\alpha ,\beta \right)&=V\left( {{s}_{i+1}};{{\theta }^{-}},\alpha \right) \nonumber \\&\quad +\left\{ A\left( {{s}_{i+1}},{{a}_{\left( i+1 \right) 1}};{{\theta }^{-}},\beta \right) -\frac{1}{|A|}\sum \limits _{{{a}'}}{A\left( {{s}_{i+1}},{{{{a}'}}_{(i+1)1}};{{\theta }^{-}},\beta \right) } \right\} , \end{aligned}$$
    where $a'$ represents the next subchannel allocation action. In the current state, each *A* is subtracted from the average of all *A* values in Eq. (21), which ensures that the expected value of the advantage function is bounded to zero and increases the stability of the output of the functions *V* and *A*. The loss function of the estimated Q-network in the dueling DQN is expressed as
    22 $$\begin{aligned} L\left( \theta \right) = {\left( {{y_i} - Q\left( {{s_i},{a_{i1}};\theta } \right) } \right) ^2}. \end{aligned}$$
  **Step 6:** For the power allocation network, the DDPG network can effectively deal with the continuous action space^44^, and the sampled target Q values are obtained for training the power allocation network
    23 $$\begin{aligned} {y_i} = {r_i} + \gamma Q\left( {{s_{i + 1}},\mu \left( {{s_{i + 1}};{\vartheta ^ - }} \right) ;{\omega ^ - }} \right) . \end{aligned}$$
    In this manner, the loss function of the estimated Q-network is given by
    24 $$\begin{aligned} L\left( \omega \right) = \frac{1}{N}\sum \nolimits _i {{{\left( {{y_i} - Q\left( {{s_i},{a_{i2}};\omega } \right) } \right) }^2}}. \end{aligned}$$
    where *N* is the batch size of the minibatch. We use an Adam optimizer to adjust $\omega$ at every time slot to minimize Equation (24). The optimizer returns a set of gradients after the minibatch sampling the experience data by
    25 $$\begin{aligned} \frac{{\partial L\left( \omega \right) }}{{\partial \omega }} = - 2\left( {{y_i} - Q\left( {{s_i}, {a_{i2}};\omega } \right) } \right) \nabla Q\left( {{s_i},{a_{i2}};\omega } \right) , \end{aligned}$$
    where $\nabla Q$ denotes the gradient of the estimated values of Q. Subsequently, the back propagation (BP) technique is employed to update the weights of the estimated Q-network.
    According to DPG theory^45^, The weight $\vartheta$ of $\mu \left( {s;\vartheta } \right)$ can be updated by the sampled policy gradient
    26 $$\begin{aligned} {\nabla _\vartheta }J(\mu )\simeq \frac{1}{N} \sum \nolimits _i{\nabla _a} Q( s,a;\omega )|_{s = s_i,a = \mu (s_i;\vartheta )} {\nabla _\vartheta } \mu ( s;\vartheta )\left| {_{s = {s_i}}} \right. . \end{aligned}$$
    **Table 1:** Setting of hyperparameters.

Hyperparameters	Values
Episode	1000
The maximum number of time slots *T*_max_	100
Updating time slots *W*	100
Minibatch size *N*	32
Discount factor *γ*	0.9
Learning rate of dueling DQN	0.01
Learning rate of actor network	0.002
Learning rate of critic network	0.004
Soft updating factor of DDPG *τ*	0.01
*ε*-greedy probability	0.9
Replay buffer memory $\left| {{{\mathscr{D}}}} \right|$	5000
Optimizer	Adam
Activation function	ReLU

**Table 2:** Setting of network parameters.

Parameters	Values
The number of antennas *N*	64
The number of users *K*	12
The number of RF chains* N*_*RF*_	4
Bandwidth	1 GHz
The number of subchannels	20
The maximum transmitted power of BS	40 W
Cell radius	200 m
Noise power density	- 174 dBm/Hz

In every *W* time slot, the weights of the networks $\mu \left( {s;{\vartheta ^ - }} \right)$ and $Q\left( {s,a;{\omega ^ - }} \right)$ are updated in a soft manner, where the weights are updated at a time,
    27 $$\begin{aligned}{} & {} {\vartheta ^ - } \leftarrow \tau \vartheta + \left( {1 - \tau } \right) {\vartheta ^ - }, \end{aligned}$$
    28 $$\begin{aligned}{} & {} {\omega ^ - } \leftarrow \tau \omega + \left( {1 - \tau } \right) {\omega ^ - }. \end{aligned}$$
    where $\tau$ is the soft update factor (considered to be relatively small). The output power allocation action of the DDPG network is the continuous transmit power of all users.
  **Step 7:** Repeat **Steps 2–6** to train the Q-network of the dueling DQN-DDPG at a certain time.

**Figure 3:**
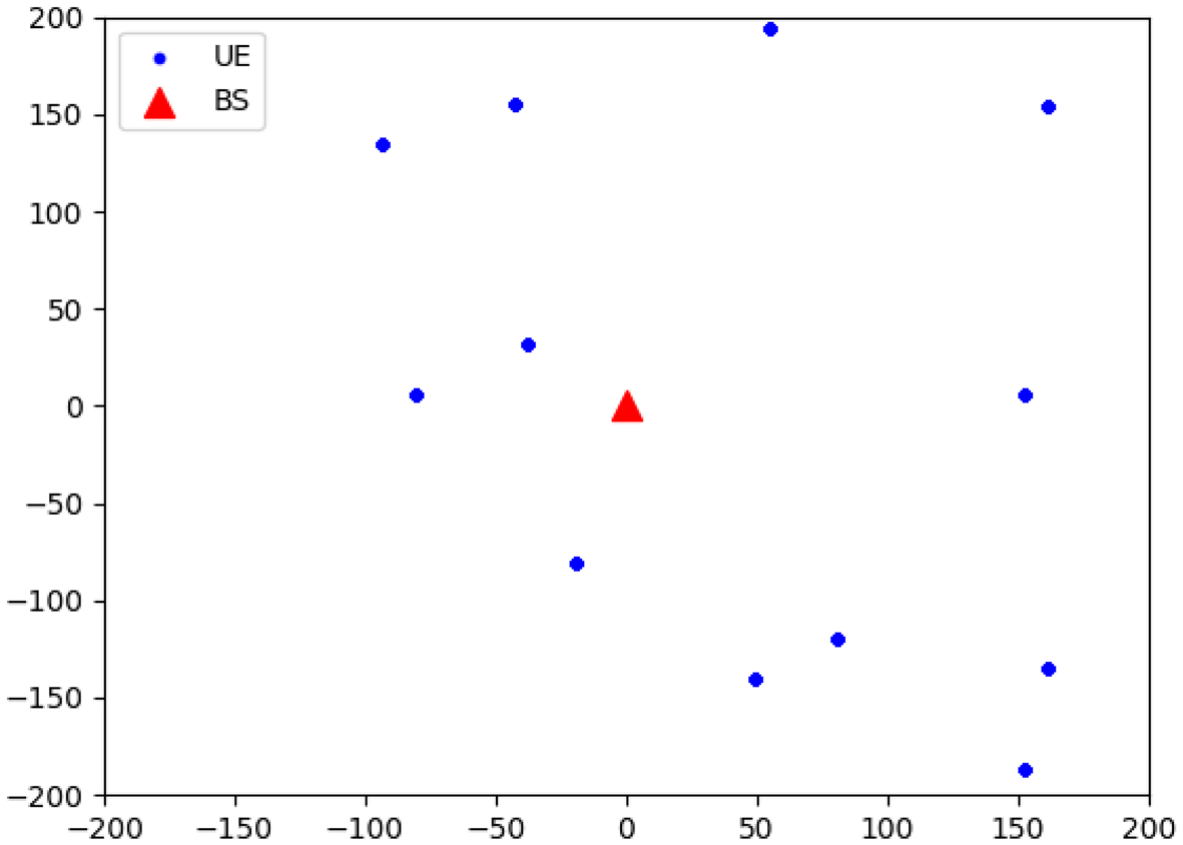
Network layout with BS and Users.

**Figure 4:**
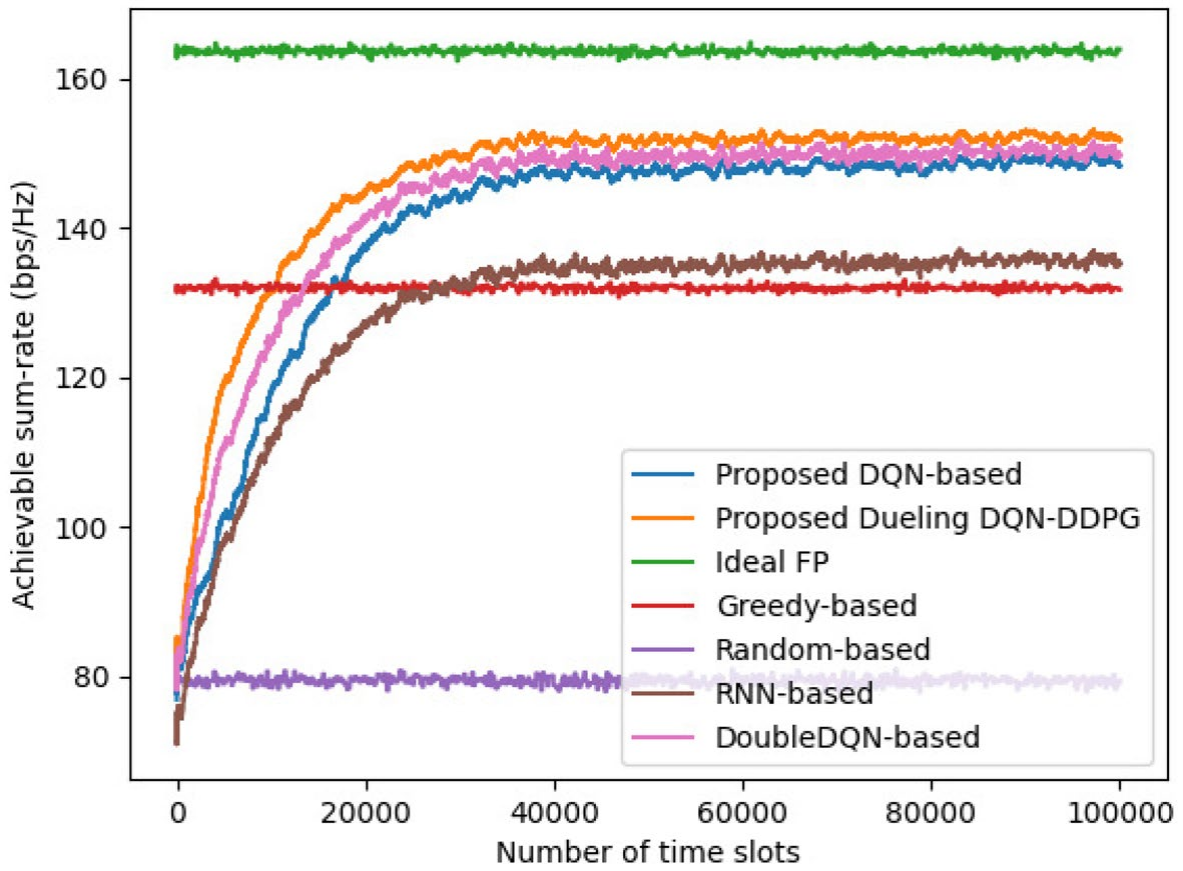
Comparison of the performance of different allocation schemes versus time slots.

**Figure 5:**
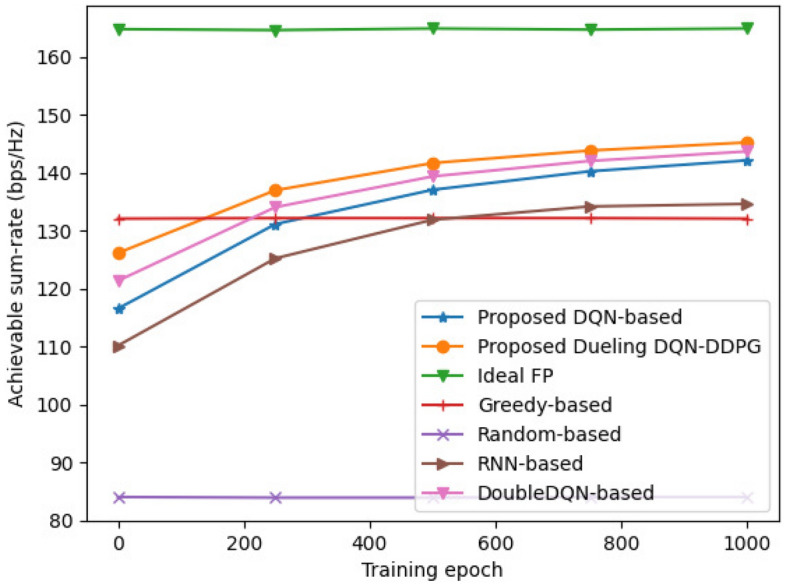
Comparison of the performance of different allocation schemes versus training epoch.

**Figure 6:**
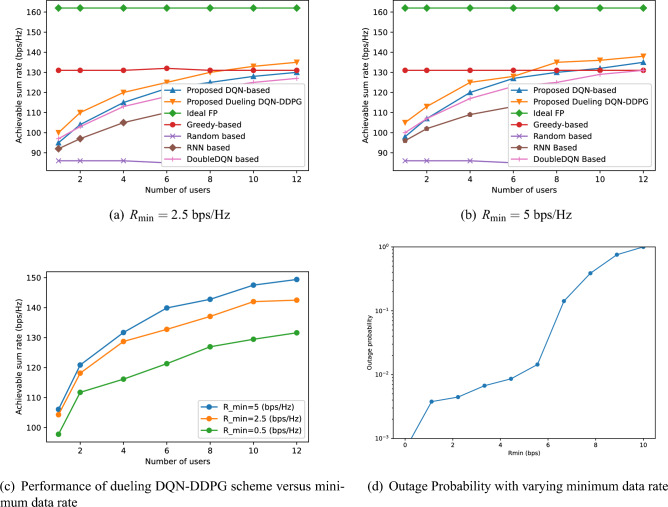
Comparison of the performance of different allocation schemes versus the number of users and outage probability with varying minimum data rate.

**Figure 7:**
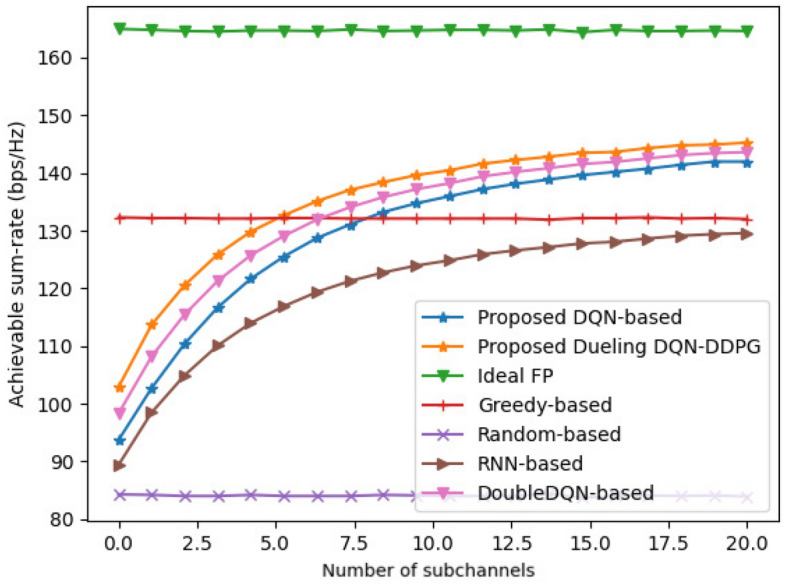
Comparison of the performance of different allocation schemes versus the number of subchannels.

**Figure 8:**
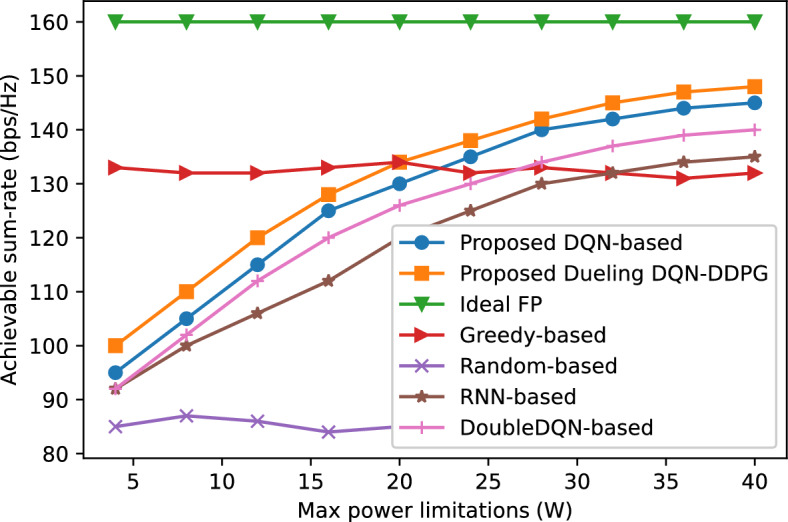
Comparison of the performance of different allocation schemes under various transmit power limitations.

The training process of the proposed scheme is shown in Algorithm 3.

In summary, our proposed framework expedites the convergence of user grouping by utilizing an advanced K-means approach, thereby mitigating multi-user interference. It incorporates a decision-making DQN for subchannel allocation, enhancing convergence rates. Additionally, the continuous power allocation facilitated by the DDPG algorithm safeguards against performance loss attributed to power quantization errors, markedly improving the system’s spectral efficiency. Compared to existing algorithms, this framework can manage resources with finer granularity, greatly amplify system adjustability and spectrum utilization, and demonstrates formidable potential in addressing resource allocation issues in 6G and future wireless communication systems.

## Simulation results

To evaluate the performance of the proposed dueling DQN-DDPG-based scheme, we compare it with several existing algorithms in this section, such as the random allocation algorithm (a baseline without optimization), the greedy allocation algorithm^46^ (a common algorithm that maximizes throughput but causes interference and unfairness), the DoubleDQN algorithm^31^ (a DRL-based algorithm that reduces Q value bias but has a discrete action space), the RNN algorithm^47^ (a neural network-based algorithm that dynamically allocates resources based on channel state and user demand), and the DQN-based algorithm in Appendix A. We also use the optimal FP algorithm^11^ (a mathematical optimization technique that can find the optimal solution of the non-convex power allocation problem but needs channel state information and has a high complexity) as a benchmark. The hardware environment of the simulation experiment platform equips i7-10700 CPU and 16GB RAM running memory, and the software platforms are Win10 and Python 3.7. All the DNNs in the proposed framework contain a fully connected hidden layer with 64 neurons and a fully connected hidden layer with 32 neurons. The rectified linear unit (ReLU) is used as the activation function,

29 $$\begin{aligned} f\left( x \right) = \max \left( {0,x} \right) . \end{aligned}$$

Tables 1 and 2 summarize the parameters used in this simulation. The network layout is shown in Fig. 3, which consists of a single small cell with a radius of 200 m that equips one BS which is located at the center of the cell with coordinates $\left( {0,0} \right)$ and is marked with a red triangle, and serves 12 users. The users represented by the blue dots randomly distribute within the cell, and their locations change randomly in each simulation.

A comparison of achievable sum-rate is presented in Fig. 4, where the number of iterations is 1000, and each iteration includes 100 time slots, then the total number of time slots is 100,000. It is apparent that the optimal FP algorithm exhibits the best performance because of the instantaneous global channel state information. In addition, the DRL-based algorithms have better performance than the RNN-based scheme because of the interaction learning ability. Specifically, the proposed dueling DQN-DDPG-based scheme requires fewer time slots than other allocation algorithms when the achievable sum-rate is same. This is because the dueling DQN can solve the slow convergence problem by splitting the value function into more detailed representations. Moreover, because the power allocation action of the proposed DDPG-based strategy can be selected continuously, it is not necessary for the BS to select the power level from the quantized power action space to obtain the current optimal joint subchannel and power allocation action at each time slot, which can solve the performance loss caused by discrete power actions. Therefore, the proposed allocation scheme can make a near-optimal allocation strategy when the network changes in real time and achieve a higher network achievable sum-rate.

Figure 5 demonstrates the impact of training epochs on the performance of different allocation schemes. The system achievable sum-rate of the neural network-based schemes (including proposed dueling DQN-DDPG, DQN-based, DoubleDQN-based, and RNN-based schemes) increases with the training epochs. This is because that the greater the number of training epochs, the more accurate the weights of the network become, enabling a better selection of the allocation strategy. As the training epochs increase to a certain number, the achievable sum-rate of the neural network-based schemes grows gradually and remains stable, because there is less new environmental information between the BS and the user to be learned. In addition, the proposed dueling DQN-DDPG scheme outperforms the other neural network-based algorithms under the same number of training epochs and requires fewer training epochs to achieve the same achievable sum-rate. Thus, the proposed scheme can achieve better performance with shorter training epochs.

Figures 6a,b depict the achievable sum-rate performance versus the number of users for different schemes, thereby illustrating how user grouping and diverse user impacts influence sum-rate performance across various allocation strategies. It is evident that the random allocation strategy has the worst performance, while the FP allocation scheme performs the best. In addition, the performance of the greedy-based, FP, and random-based allocation schemes maintain stable as the number of users increases, while the achievable sum-rate of the neural network-based schemes gradually increases and stabilizes. This is because that the neural network-based schemes obtain the optimal resource allocation strategy by adjusting the allocation actions based on feedback through BS interaction with users. When the number of users is small, e.g. less than 3, BS obtains a small amount of data which leads to a low achievable sum-rate. However, the BS expands the interaction with the users as the users’ numbers increase and obtains the optimal resource allocation scheme for more users. Although the performance of all schemes improves as the minimum data rate increases, we can see that our proposed dueling DQN-DDPG based scheme is superior to other neural network-based schemes and is closer to the optimal FP allocation algorithm. This is because that the subchannel and power action spaces are efficiently handled by dueling DQN and DDPG, making better resource allocation decisions when the network changes. Moreover, as the user persistently exceeds the number of RF, user grouping effectively elevates the multiplexing gains and reduces interference, thereby significantly impacting the performance. As a result, the achievable rate of the proposed algorithm surpasses that of other related algorithms. However, as the number of users continues to increase beyond the capacity of RF, inter-user interference becomes the primary factor limiting further enhancements in system performance. Consequently, the system performance tends to stabilize. Figure 6c illustrates the effects of the number of users and minimum achievable rate threshold on the proposed algorithm. The performance of the proposed algorithm initially rises and then reaches saturation as the number of users increases. This is attributed to the fact that excessive users will result in the intensification of multi-user interference, which impedes the further enhancement of the system rate. Moreover, a higher minimum achievable rate threshold can encourage the proposed algorithm to choose a more optimal subchannel and power allocation strategy, which consequently improves the system rate. Thus, for a fixed number of users, the proposed algorithm achieves a higher rate when the minimum achievable rate threshold is higher. The unexpected enhancement in overall rate performance observed may be attributed to the capacity and bandwidth of the base station: within the parameters of our study, the base station possesses sufficient transmission capabilities and bandwidth to support elevated $R_{\min }$ values, thereby ensuring the minimum rate requirements of users are met. The strategy employed by the DRL algorithm reveals a refined approach to resource allocation, which maintains high total system throughput even as $R_{\min }$ is increased. This strategy enables the algorithm to navigate more stringent constraints effectively, utilizing resource management and user scheduling to uncover and capitalize on optimization opportunities previously unexplored. However, as the minimum rate threshold increases, resource allocation becomes more concentrated, leading to a higher likelihood of service interruption for users at the periphery or those in adverse transmission conditions. Hence, the influence of minimum rate requirements on the probability of interruptions is shown in Fig. 6d. The simulation outcomes indicate a direct correlation between the heightened minimum rate thresholds and the increased probability of interruptions. Specifically, surpassing a minimum rate requirement of 5 bps in wireless communication systems escalates the chances of data transmission interruptions. This suggests that meeting higher rate demands compromises the system’s reliability, consequently heightening the risk of disruptions in data transmission.

We evaluate the effects of the number of subchannels on the performance of different schemes in Fig. 7. The achievable sum-rate of the neural network-based schemes increases as the number of subchannels increases. Specifically, the action spaces of the DRL-based schemes become prohibitive when the number of subchannels becomes large enough; thus, the convergence speed of these schemes becomes slow. As the number of subchannels increases, the transmitting data on each channel decreases and the total SINR per user increases, then the BS as an agent can obtain more network state information. Therefore, the proposed scheme can make more suitable actions according to the changes of the network, and its performance is greater than that of other neural network-based allocation algorithms and is more nearly to that of optimal FP algorithm. Furthermore, the optimization of user grouping algorithms has significantly enhanced spectral reuse gain and reduced interference. The increase in the number of subchannels has also augmented the flexibility of resource allocation, enabling DRL algorithms to allocate power and spectral resources with greater agility. The benefits of channel diversity have been manifested, thus, with an increased number of subchannels, DRL algorithms excel at selecting subchannels with superior conditions, thereby elevating the overall performance of the system.

We evaluate the effect of the maximum power constraint on the spectral efficiency of different schemes. In Fig. 8, it is clear that the random-based allocation scheme has the worst performance due to its randomness. The greedy-based allocation scheme has a relatively good performance with a small maximum power constraint, e.g., less than 10 W, while the DRL-based schemes outperform the greedy-based scheme as the maximum power constraint increases. The proposed dueling DQN-DDPG-based scheme exhibits better performance than other neural network-based schemes and achieves approximately 88% of the optimal FP algorithm when the maximum power constraint is ${P_{\max }} = 40$ W. Contrary, the DoubleDQN-based, proposed DQN-based, greedy-based, RNN-based and random-based schemes achieve 87%, 86%, 81%, 79% and 51% of the optimal FP algorithm, respectively.

## Conclusion

In this paper, to reduce the multi-user interference and co-channel interference with high convergence speed of mmWave massive MIMO-NOMA system, a new enhanced K-means user grouping algorithm is proposed. Furthermore, we use the adaptive decision-making DRL to solve the non-convex and NP-hard problem in joint subchannel and power allocation optimization. Specifically, a dueling DQN-DDPG-based framework is proposed, where the dueling DQN is designed to accelerate the convergence speed of the subchannel allocation network and the proposed continuous DDPG algorithm solves the problems of power allocation performance loss caused by the power quantization and the slow convergence of traditional DL-based methods. Our proposed schemes effectively accelerate the convergence speed and substantially improve the achievable sum-rate performance compared to that of some existing resource allocation algorithms.

Although the proposed DRL-based framework substantially improves the grouping efficiency and resource allocation in mmWave massive MIMO-NOMA systems, there exist inherent challenges and complexities necessitating further study, such as the management of interference, intricacy of resource allocation, difficulties in channel estimation and feedback, and the issue of energy efficiency. Our subsequent work will focus on crafting sophisticated models and plans not only to overcome these constraints but also to fully exploit the capabilities of mmWave massive MIMO-NOMA systems for the enhancement of forthcoming wireless networks. Future research will employ advanced deep reinforcement learning techniques, particularly PPO and A3C algorithms, to enhance next-generation wireless networks. PPO will ensure stable training and optimize resource allocation, while A3C will improve user grouping and resource management through asynchronous learning, collectively boosting the performance and efficiency of mmWave massive MIMO-NOMA systems.

## Data Availability

The datasets used and/or analysed during the current study available from the corresponding author on reasonable request.

## Appendix A The proposed DQN-based algorithm

We propose to use the DQN network to solve the problem of resource allocation to receivers in each user group. The current state of the network ${s_t}$ which is obtained by the agent through interaction with the network is used as an input to the DQN network. After the agent takes action ${a_t}$ from action space ${{{\mathscr {A}}}}$, the DQN network outputs the Q value. Moreover, the agent can receive an instant reward ${r_t}$ from the network and the next state ${s_{t + 1}}$. These components are defined as follows:

1. *States of DQN*: State ${s_t} \in {{{\mathscr {S}}}}$ needs to reflect the current SINR of all users, and the states at the *t*th time slot are also defined as Equation (13).
2. *Actions of DQN*: The output of the DQN is discrete, and action space ${{{\mathscr {A}}}}$ contains all the available subchannel allocation factors ${x_{l.m.k}}\left( t \right)$ and the allocated power ${P_{l,m,k}}\left( t \right)$. The actions at the *t*th time slot are also defined as Equation (14), where $a_{t1}=\{{x_{1,1,1}}(t),\ldots ,x_{l,m,|{\Omega _m}|}(t),\ldots , x_{L,{N_{RF},|{\Omega _{{N_{RF}}}}|}}(t)\}$, $a_{t1}\in {{{{\mathscr {A}}}}_1}$; ${a_{t2}} = \{ {{P_{1,1,1}}( t ), \ldots ,{P_{l,m,| {{\Omega _m}} |}}( t ), \ldots ,{P_{L,{N_{RF}},| {{\Omega _{{N_{RF}}}}} |}}( t )} \}$, ${a_{t2}}\in {{{{\mathscr {A}}}}_2}$. ${{{{\mathscr {A}}}}_1}$ is the subchannel allocation action space, ${{{{\mathscr {A}}}}_2}$ is the power allocation action space, and the size of the total action space is $2L \times K$. We use a multi-DQN architecture^33^ that can work in a synchronous and distributed manner, in which one DQN can determine the power allocation policy for one user. Moreover, the number of selected power actions is reduced from ${E^K}$ to $E \times K$ after using the multi-DQN architecture, improving the efficiency of the network. Therefore, the actions of power allocation can be rewritten as
  30 $$\begin{aligned} a_{t2} = \{ a_{t2}^{1,1},a_{t2}^{1,2}, \ldots ,a_{t2}^{m,1}, \ldots ,a_{t2}^{m,\left| {{\Omega _m}} \right| }, \ldots ,a_{t2}^{{N_{RF}},1}, \ldots , a_{t2}^{{N_{RF}},\left| {{\Omega _{{N_{RF}}}}} \right| } \}, \end{aligned}$$
  where the power allocation action $a_{t2}^{m,\left| {{\Omega _m}} \right| }= v \times \frac{{{P_{\max }}}}{E}$, $v \in \left\{ {1,2, \ldots , E} \right\}$ is selected by the $\left| {{\Omega _m}} \right|$th DQN in the *m*th group at the *t*th time slot. Since only *K* users need power allocation, ${P_{l,m,k}}\left( t \right)$ can be rewritten as
  31 $$\begin{aligned} {P_{l,m,k}}\left( t \right) = \left\{ {\begin{array}{ll} {0,} &{} {{x_{l,m,k}}\left( t \right) = 0} \\ {{P_{l,m,k}}\left( t \right) ,} &{} { {x_{l,m,k}}\left( t \right) = 1} \\ \end{array}} \right. . \end{aligned}$$
3. *Reward function of DQN*: The reward function Equations (16) and (17) are used to train the model of the multi-DQN resource allocation scheme.
4. *Training of DQN*: The experience replay mechanism is employed to train the proposed model. The specific training steps are as follows:
  - **Step 1:** Initialize the states, actions, weight update interval, and replay memory buffer. Then, networks $Q\left( {{s_t},{a_t};\theta } \right)$ and $Q\left( {{s_{i + 1}},{a_{i + 1}};{\theta ^ - }} \right)$ are initialized with random weights $\theta$ and ${\theta ^ - } \leftarrow \theta$, respectively.
  - **Step 2:** At the beginning of each step (episode), BS obtains the initial state ${s_1}$ from the mmWave massive MIMO-NOMA system. The $\epsilon$-greedy strategy is employed for the DQN network to select the subchannel allocation action ${a_{t1}}$.
  - **Step 3:** The *K* users are divided into ${N_{RF}}$ groups through the proposed user grouping algorithm, and the *K* DQN architectures are selected for the activation of the power allocation network. Each DQN architecture corresponds to the power allocation policy for one user. The network state ${s_1}$ inputs to the power allocation DQN network. The DQN outputs the selected power allocation action ${a_{t2}}$ by $\epsilon$-greedy strategy after obtaining the estimated Q value. It is worth noting that these DQN architectures have the same replay memory and fixed target network methods and structures, while the weights of these networks are different.
  - **Step 4**: The agent obtains the returned reward ${r_t}$ and receives the next state ${s_{t + 1}}$ of the network after executing a joint action ${a_t} = \left\{ {{a_{t1}},{a_{t2}}} \right\}$.
  - **Step 5:** During the training of the DQN, the experience replay mechanism is used to store the experience $\left( {{s_t},{a_{t1}},{r_t},{s_{t + 1}}} \right)$ in ${{{\mathscr {D}}}}$ at each time slot. To ensure that the training samples of the DQN are independent and uncorrelated, the minibatch of size *N* is randomly sampled from memory ${{{\mathscr {D}}}}$. Then, the minibatch training of the DQN is performed.
    The target Q-network generates the target Q value for the subchannel allocation network as
    32 $$\begin{aligned} {y_i} = {r_i} + \gamma \mathop {\max }\limits _{{a_{\left( {i + 1} \right) 1}} \in {{{{\mathscr {A}}}}_1}} Q\left( {{s_{i + 1}},{a_{\left( {i + 1} \right) 1}};{\theta ^ - }} \right) . \end{aligned}$$
    The objective of the training is to make the prediction error between the estimated and the target Q values infinitely close to zero. Therefore, we define a loss function for the prediction error
    33 $$\begin{aligned} L\left( \theta \right) = {\left( {{y_i} - Q\left( {{s_i},{a_{i1}};\theta } \right) } \right) ^2}. \end{aligned}$$
    An Adam optimizer is used to adjust $\theta$ at every time slot to minimize Equation (33). The optimizer returns a set of gradients after the minibatch sampling the experience data by calculating
    34 $$\begin{aligned} \frac{{\partial L\left( \theta \right) }}{{\partial \theta }} = - 2\left( {{y_i} - Q\left( {{s_i},{a_{i1}};\theta } \right) } \right) \nabla Q\left( {{s_i},{a_{i1}};\theta } \right) . \end{aligned}$$
    The weights of the estimated Q-network are updated using the BP technique.
    The power allocation network uses the same DQN training method as the subchannel allocation network. Thus, the target Q value can be calculated by
    35 $$\begin{aligned} {y_i} = {r_i} + \gamma \mathop {\max }\limits _{{a_{\left( {i + 1} \right) 2}} \in {{{{\mathscr {A}}}}_2}} Q\left( {{s_{i + 1}},{a_{\left( {i + 1} \right) 2}};{\theta ^ - }} \right) . \end{aligned}$$
    Then, the corresponding loss function is defined as
    36 $$\begin{aligned} L\left( \theta \right) = {\left( {{y_i} - Q\left( {{s_i},{a_{i2}};\theta } \right) } \right) ^2}. \end{aligned}$$
    Moreover, we use the Adam optimizer to minimize the loss function Equation (36). Finally, the weights of the target Q-network of the these DQN architectures are updated by coping with the weights of their corresponding estimated Q-network in every *W* time slots.
  - **Step 6:** Repeat **Steps 2–5** to train the Q-network at a certain time.

This completes the description of the proposed DQN-based algorithm.
